# Blood-Flow Restricted Warm-Up Alters Muscle Hemodynamics and Oxygenation during Repeated Sprints in American Football Players

**DOI:** 10.3390/sports7050121

**Published:** 2019-05-21

**Authors:** Jean-François Fortin, François Billaut

**Affiliations:** Department of Kinesiology, Laval University, Quebec, QC G1V 0A6, Canada; jf_9@hotmail.com

**Keywords:** warm-up, blood-flow restriction, pre-conditioning, repeated-sprint ability, team sports, muscle oxygenation

## Abstract

Team-sport athletes and coaches use varied strategies to enhance repeated-sprint ability (RSA). Aside from physical training, a well-conducted warm-up enhances RSA via increased oxidative metabolism. Strategies that impede blood flow could potentiate the effects of a warm-up due to their effects on the endothelial and metabolic functions. This study investigated whether performing a warm-up combined with blood-flow restriction (WFR) induces ergogenic changes in blood volume, muscle oxygenation, and RSA. In a pair-matched, single-blind, pre-post parallel group design, 15 American football players completed an RSA test (12 × 20 m, 20 s rest), preceded by WFR or a regular warm-up (SHAM). Pressure was applied on the athletes’ upper thighs for ≈15 min using elastic bands. Both legs were wrapped at a perceived pressure of 7 and 3 out of 10 in WFR and SHAM, respectively. Changes in gastrocnemius muscle oxygen saturation (S_m_O_2_) and total hemoglobin concentration ([THb]) were monitored with near-infrared spectroscopy. Cohen’s effect sizes (ES) were used to estimate the impact of WFR. WFR did not clearly alter best sprint time (ES −0.25), average speed (ES 0.25), total time (ES −0.12), and percent decrement score (ES 0.39). While WFR did not meaningfully alter average S_m_O_2_ and [THb], the intervention clearly increased the maximum [THb] and the minimum and maximum S_m_O_2_ during some of the 12 sprint/recovery periods (ES 0.34–1.43). Results indicate that WFR positively alters skeletal muscle hemodynamics during an RSA test. These physiological changes did not improve short-term RSA, but could be beneficial to players during longer activities such as games.

## 1. Introduction

In American football, as in most team sports, athletes typically repeat short-duration, high-intensity bouts of movement such as sprinting, interspersed with brief periods of low-intensity activity [1]. As such, repeated-sprint ability (RSA) is an important determinant of performance in American football, especially for the skilled players (i.e., wide receivers, running backs, quarterbacks, tight ends, defensive backs, and linebackers) who execute the greatest numbers of sprints throughout a match [1]. Research has demonstrated that improving RSA through physical training has clear benefits for team-sport athletes [2,3], with a likely advantageous transfer to the playing field [4,5].

Athletes and coaches use varied interventions to enhance RSA, with the goal of delaying the onset of fatigue. A large portion of the inability to reproduce performance across sprint repetitions can be traced to limitations in energy supply and metabolite accumulation [6]. Therefore, improving the contribution of the oxidative metabolism generally mitigates the influence of these limiting factors, and enhances the ability to resist fatigue during repeated-sprint exercise (RSE) (for a review, see [6]). Several physiological adaptations related to greater oxidative phosphorylation such as higher maximal oxygen consumption (VO_2_max) [7,8,9,10,11] and faster oxygen uptake kinetics [12,13,14] have been associated with greater RSA. For example, participants with a greater VO_2_max are more able to maintain power output during RSE [15], especially during the latter sprints when participants may reach their VO_2_max [13], and athletes with faster VO_2_ kinetics usually get a shorter cumulated time and a lower relative decrease in speed during RSE [13]. In addition, it seems that between-sprint muscle reoxygenation capacity may influence the ability to resist fatigue [16], with faster reoxygenation rate being correlated to lower performance decrement [17].

Aside from physical training, a well-conducted warm-up may also enhance RSA by acutely improving skeletal muscle VO_2_ (mVO_2_) [18] and VO_2_ kinetics [12]. Since every athlete conducts some form of warm-up before training or competition, it is a performance optimization opportunity not to be neglected. The majority of the warm-up ergogenic effects on performance have been attributed to elevated muscle temperature (T_m_) [19]. These mechanisms include vasodilation of blood vessels and increased muscle blood flow [20]. The improved muscle oxygenation may accelerate VO_2_ kinetics provided that exercise intensity is high enough so the VO_2_ kinetics is limited by O_2_ delivery [21,22,23]. As a result, vigorous active warm-up, but not passive warm-up, has been shown to enhance VO_2_ kinetics [12,24,25,26,27,28,29,30]. Therefore, one may argue that a warm-up specifically designed to enhance O_2_ delivery and mVO_2_ should provide an ergogenic potential.

In this perspective, strategies that acutely restrict limb blood flow promote vasodilation of muscle blood vessels, increase blood flow, and ultimately improve O_2_ delivery to render many organs, including skeletal muscles, more resistant to subsequent ischemic/hypoxic events, similar to those observed during high-intensity exercise [31]. When used at rest before exercise, such strategies can improve maximal performance in various exercise modes when the oxidative system is fully taxed [31]. During exercise, the so-called blood-flow restriction (BFR) technique also allows manipulating blood flow to skeletal muscles [32]; however, the impact on acute performance is still unclear. The pressure applied to the limbs during BFR is sufficient to impede the venous outflow but maintains approximately 50–80% of the arterial inflow [32]. BFR potentially leads to the acute release of nitric oxide (NO) [33], as well as adenosine [34], two substances known for their vasodilatory properties. Not surprisingly then, BFR has been robustly demonstrated to enlarge the maximal artery dilatation [35], increase reactive hyperemia [36,37], and elevate muscle oxygenation post-BFR [36]. These BFR-induced acute adaptations of the endothelial and metabolic functions mimic some of the mechanisms of a warm-up, and could thereby potentiate the effects of a warm-up on a subsequent performance.

Therefore, the aim of this study was to assess the impact of a warm-up combined with blood-flow restriction (WFR) on changes in local blood volume, muscle oxygenation, and performance during a subsequent RSA test. Based on the above-cited mechanisms of blood-flow restriction, it was expected that WFR would improve RSA via greater regional blood volume and enhanced O_2_ availability.

## 2. Materials and Methods

### 2.1. Participants

In all, 16 male athletes were recruited from the competitive roster of the 2016 Laval University Canadian football team (age, 21.0 ± 1.8 years; height, 188.2 ± 18.9 cm; weight, 82.1 ± 2.8 kg). The group of participants was composed of offensive and defensive players, playing either quarterback, running back, receiver, linebacker, or defensive back. Kickers and linemen were excluded because these athletes are usually less accustomed to repeating sprints. All participants were free of any medical conditions as stated on a self-report health questionnaire. The players selected were training regularly 5–6 d·wk^−1^ in the off-season strength and conditioning program during this study and were all in good physical condition. To ensure methodological validity, they were asked to avoid vigorous exercise, alcohol, caffeine, energy drinks, and any supplements that could alter blood flow for 24 h before the tests. Participants were fully informed about the purposes, benefits, and risks associated with the experimental procedures before giving written informed consent to their participation in this study. Ethical approval was obtained from the Laval University ethics committee and the experiment was conducted in accordance with the Declaration of Helsinki.

### 2.2. Experimental Design

Athletes visited the laboratory on three occasions during which all testing procedures were performed by the same investigator. The main goal of the first visit (V1) was to collect baseline data for future comparisons. During this session, height, weight, and thigh volume were measured. Thigh volume was calculated using the frustrum method that has been used in previous studies [38,39]. The method is based on the assumption that the thigh approximates in shape to a truncated cone, or frustrum, so the thigh volume is calculated as the volume of a truncated cone:

V = π/3 · h · (R^2^ + Rr + r^2^), (1)

where, h = √ h’^2^ − (R − r)^2^, h’ being the length of the thigh based on surface measurement, while R and r are radii calculated from the greatest and the smallest circumference of the thigh, respectively. To avoid complications related to hemodynamics alterations caused by WFR, every athlete’s resting heart rate and blood pressure were monitored. Participants then completed a standardized active warm-up consisting of two sections of dynamic exercises that the players were used to doing before every off-season speed and agility workout. The first section lasted approximately 15 min (14 min 34 s ± 1 min 15 s). It included dynamic stretches and various activation exercises for the posterior chain and the ankle stabilizing muscles before ending with a submaximal 20 m sprint. The second section was shorter (4 min 46 s ± 0 min 27 s) and comprised vigorous movements of progressive intensity. It ended with two 20 m sprints, the last one being a maximum effort. Both sections were separated by a 5 min pause during which thigh volume was measured again. At the end of the active warm-up, participants rested standing for two minutes before completing the RSA test (see below for procedures). The average percent decrement score (%S_DEC_) from this test was calculated as described below and used along with best sprint time (S_BEST_) and total time (S_TOTAL_) to pair match athletes and assign them to one of the two experimental groups (WFR or regular warm-up (SHAM)). There was no clear difference in group means for all three performance parameters tested during the first visit.

During the second visit, participants completed a familiarization of the compression method. In both groups, powerlifting elastic knee wraps (Hyperforce, Cantley, QC, Canada; width: 7.5 cm, length: 200 cm) were used to apply pressure to the thighs. This technique has been described elsewhere [40,41]. Before wrapping, the perceived pressure scale was presented to the participants. It was explained that a 0 out of 10 pressure meant no pressure, a 7 out of 10 pressure meant moderate pressure with no pain, and a 10 out of 10 pressure was described as intense pressure with pain. The investigator made sure that the explanations were understood by the participants and they reviewed the scale until full comprehension in every case. Then, the elastic wraps were applied at the proximal end of both lower limbs (at the top of the thigh, near the inguinal crease). The length of the elastic wraps allowed for several wraps around the thigh. After each wrap, the participants were asked to rate the perceived pressure according to the 0-to-10 rating scale. Under both conditions, the first wrapping process continued until an intense pressure (9 or 10 out of 10) was reached after which point the wraps were promptly removed. It was explained that this high pressure level should not be reached in any further procedures. The participants were in fact introduced to intense pressure to allow them to determine with greater precision the perceived pressure level in subsequent wrapping processes. After a short pause to allow the participants to rest their legs, the same investigator started the second wrapping process. In the SHAM group, the wrapping ended when a light pressure (3 out of 10) was attained. In the WFR group, wrapping ceased when a moderate pressure was reached (7 out of 10). Such moderate pressure has been shown to be effective to fully occlude venous, but not arterial, blood flow [41]. To minimize any placebo effect, participants were told that the study purpose was to compare the impact of two different pressure levels that could both alter performance. The participants then completed the first section of the active warm-up (≈15 min) with the wraps still in place around their thighs. The RSA test was not administered and no data were collected during that visit.

At the beginning of the third visit (V3), thigh volume was measured at rest when participants arrived at the track. Then the participants had to complete both sections of the active warm-up as performed during the first visit, except this time they had to complete the first section with either the light (SHAM) or the moderate (WFR) pressure. During the five-minute pause that separated the two sections of the warm-up, the wraps were removed and thigh volume was measured again to assess the potential limb girth increase caused by the compression. After the warm-up, the participants rested standing for two minutes and completed the RSA test.

### 2.3. Repeated-Sprint Ability Test

To avoid any potential effect of the wind and weather, all the RSA tests were performed on the interior track and field complex of Laval University. The test protocol consisted of twelve 20 m running sprints interspersed with 20 s of active recovery. During the recovery periods, participants had to jog back to the starting line, so all the sprints were run in the same direction. Because one of the goals of the study was to investigate the influence of WFR on the ability to resist fatigue during repeated sprints, it was essential that the test protocol elicit a significant level of fatigue. According to several field observations made by the team’s strength and conditioning coach at the time, 20 s of passive recovery between efforts did not lead to a significant decrease in speed throughout twelve 20 m sprints. Therefore, the recovery periods were made active in order to slightly increase fatigue and elicit performance decrement. Sprints were initiated with the participants’ dominant leg, from a two-point stance position. Participants were instructed to sprint as fast as possible and to maintain maximal effort from the first sprint to the last. The starting signal for each sprint was given verbally and strong verbal encouragement was provided throughout the test. Following the last sprint, participants performed a self-paced cool down.

Wireless electronic timing gates (Smartspeed Lite timing gates system, Fusion Sport, Chicago, IL, USA) were used to measure sprint times. These gates have a typical error of measurement of less than 0.03 s and a coefficient of variation (CV) of less than 1% over distances around 30 m. The gates were connected to a wireless portable scoreboard, so the participants could compare their own performance between each sprint (Smartscore wireless scoreboard, Fusion Sport, Chicago, IL, USA). The fastest sprint of the series was analyzed for time (S_BEST_, s) and speed (S_SPD_, m·s^−1^), whereas S_TOTAL_ was calculated as the sum of the time values from all sprints (S_n_, where n = 1:12). Total time for sprint clusters 1–3 (S_1–3_), 4–6 (S_4–6_), 7–9 (S_7–9_), and 10–12 (S_10–12_) were also calculated. The percent decrement score across the twelve sprints was determined as the percent difference between total and ideal time, where ideal time represents the number of sprints multiplied by S_BEST_ [42]:

%S_DEC_ = [(S_1_ + S_2_ + S_3_ + … + S_12_)/S_BEST_ × 12) − 1] × 100. (2)

### 2.4. Near-Infrared Spectroscopy Measurements

During the first and third visits, muscle blood volume and oxygenation were monitored using portable near-infrared spectroscopy (NIRS) devices (MOXY, Fortiori Design LLC, Hutchinson, MN, USA). The MOXY monitor is a self-contained, compact (61 × 44 × 21 mm) and lightweight (48 g) NIRS system. It works by emitting near-infrared light into the muscle tissue (wavelength, 630–850 nm) and detecting some of the light after it has returned to the surface. The reflected near-infrared light is collected by two optical detectors located at a distance of 12.5 and 25 mm from the source. Because oxygenated and deoxygenated hemoglobin have different absorbance spectra, they reflect light differently. Consequently, the MOXY device can report changes in both total tissue hemoglobin concentration ([THb]) and oxygenated hemoglobin expressed as a percentage of the total hemoglobin (S_m_O_2_).

The MOXY monitors were fixed on the lateral head of the right and left gastrocnemius muscle belly. To account for skin and adipose tissue thickness covering the muscle, skinfold thickness was measured at the sites of application of the NIRS devices (6.9 ± 1.7 mm) using a Harpenden skinfold caliper (Harpenden Ltd., British Indicators Ltd, West Sussex, Great Britain). The skinfold thickness was less than half the distance between the emitter and the farthest detector (i.e., 25 mm) in every case. This thickness is adequate to let the near-infrared light travel into muscle tissue [43]. The skin was cleaned with an alcohol pad and the devices were held in place via double-sided stick disks and tape. Black bandage was used to cover the devices to reduce the intrusion of extraneous light. The locations of the MOXY monitors were marked with an indelible pen and participants were instructed to darken the marks if they started to fade before the subsequent visit.

The NIRS data were acquired at 2 Hz. The raw muscle O_2_ saturation (S_m_O_2_, %) and total hemoglobin concentration ([THb], µmol) signals were treated using a rolling average to reduce the noise created by movement. During exercise, S_m_O_2_ represents the balance between O_2_ delivery to and extraction by the muscle, while [THb] serves as an indicator of local blood volume. Baseline values of [THb] and S_m_O_2_ were taken for 1 min at rest before completion of the active warm-up during visits 1 and 3. All the values collected afterwards were normalized and expressed as a percentage of the respective baseline values. Average values of [THb] and S_m_O_2_ were calculated for the first section of the warm-up and the entire test protocol. Minimum values of S_m_O_2_ and maximum values of [THb] for each period (sprint + subsequent recovery) of the RSA test were examined to assess physiological disturbances. Finally, maximum values of S_m_O_2_ recorded for each period were also examined due to the close relationship between muscle reoxygenation capacity and performance [17].

### 2.5. Statistical Analyses

Data in the text and figures are reported as mean ± standard deviation (SD) or percentage changes between visit 3 and visit 1 (ΔV3–V1). The WFR-SHAM differences were analyzed using Cohen’s effect size (ES) ± 90% confidence limits, and compared to the smallest worthwhile change (0.2 multiplied by the between-participant SD) [44,45]. Threshold values for Cohen’s ES statistic were >0.2 small, >0.5 moderate, and >0.8 large. Using mechanistic inferences, qualitative probabilistic terms for benefit were assigned to each effect using the following scale: <0.5%, most unlikely; 0.5% to 5%, very unlikely; 5% to 25%, unlikely; 25% to 75%, possibly; 75% to 95%, likely; 95% to 99.5%, very likely; >99.5%, almost certainly. If the chance of having better/greater and poorer/lower performances or physiological variables were both >5%, the effect was deemed “unclear” or “unmeaningful” [44,45]. We used this qualitative approach because traditional statistical approaches often do not indicate the magnitude of an effect, which is typically more relevant to athletic performance than any statistical effect [45].

## 3. Results

Of the 16 participants recruited, 15 completed the entire protocol and were included in the analysis (SHAM, n = 6; WFR, n = 9). The only athlete who could not complete the study suffered an injury during an off-season workout a few days before his first scheduled visit. All participants from the WFR group tolerated the compression procedures without particular issues or complications.

### 3.1. Morphological Measure

Thigh volumes measured pre- and post-compression during V3 in SHAM and WFR are displayed in Table 1. WFR produced a possible small effect on thigh volume compared to SHAM (pre-post difference: SHAM 0.1 ± 1.4 dL; WFR 1.7 ± 0.6 dL; ES 0.21 ± 0.15; chances of ↗/=/↘ values with WFR 57/43/0%).

### 3.2. Performance Parameters

**Figure 1:**
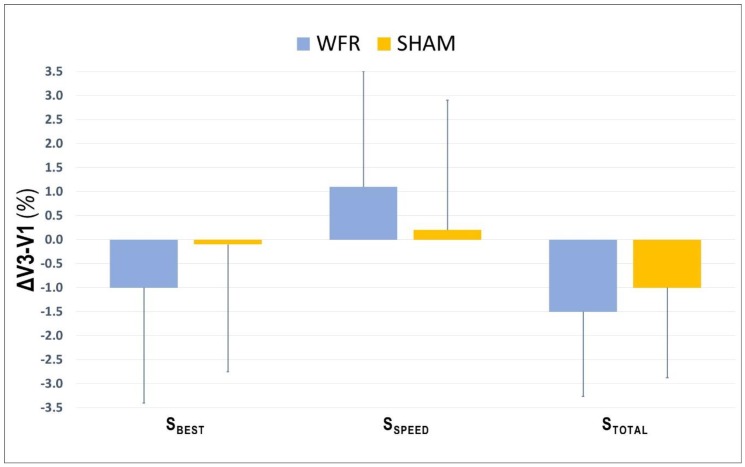
Changes from V1 in performance variables collected during V3 under SHAM and WFR conditions. Note: Data are expressed in percentage changes from V1 and presented as means ± SD.

**Figure 2:**
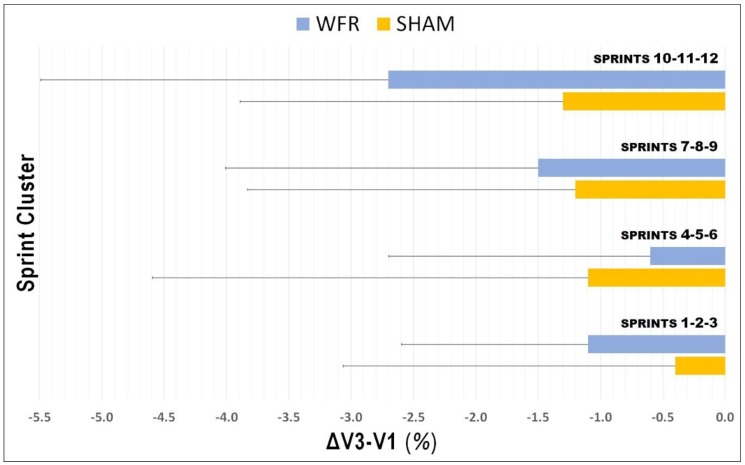
Changes in times pooled by sprint clusters from V1 to V3 under SHAM and WFR conditions. Note: Data are expressed in percentage changes from V1 and presented as means ± SD.

The average values for S_BEST_ and S_TOTAL_ were improved at V3 compared with V1, in both SHAM (S_BEST_: V1, 3.065 ± 0.128 s; V3, 3.058 ± 0.091 s; S_TOTAL_: V1, 39.687 ± 1.336 s; V3, 39.280 ± 1.663 s) and WFR (S_BEST_: V1, 3.049 ± 0.080 s; V3, 3.018 ± 0.106 s; S_TOTAL_: V1, 39.785 ± 1.649 s; V3, 39.182 ± 1.752 s). However, no clear difference was observed between groups for S_BEST_ (ΔV3−V1: SHAM −0.1 ± 2.7% vs. WFR −1.0 ± 2.4%; ES −0.25 ± 0.72; 14/31/57%), S_SPD_ (ΔV3−V1: SHAM 0.2 ± 2.7% vs. WFR 1.1 ± 2.4%; ES 0.25 ± 0.72, 55/31/14%), and S_TOTAL_ (ΔV3−V1: SHAM −1.0 ± 1.9% vs. WFR −1.5 ± 1.8%; ES −0.12 ± 0.45; 11/51/38%) (Figure 1). When times were pooled by sprint clusters, the differences between conditions were still unclear, despite a tendency for improvements with WFR in three of the four clusters (Figure 2). Notably, the WFR-SHAM difference was the greatest in cluster S_10_−S_12_ (ΔV3−V1: SHAM −1.3 ± 2.6% vs. WFR −2.7 ± 2.8%; ES −0.25 ± 0.46; 6/37/57%). Finally, data indicated that WFR had no clear impact on %S_DEC_ (ΔV3−V1: SHAM −0.9 ± 4.0% vs. WFR −0.5 ± 2.2%; ES 0.39 ± 1.74; 58/15/27%).

### 3.3. Muscle Blood Volume and Oxygenation

**Figure 3:**
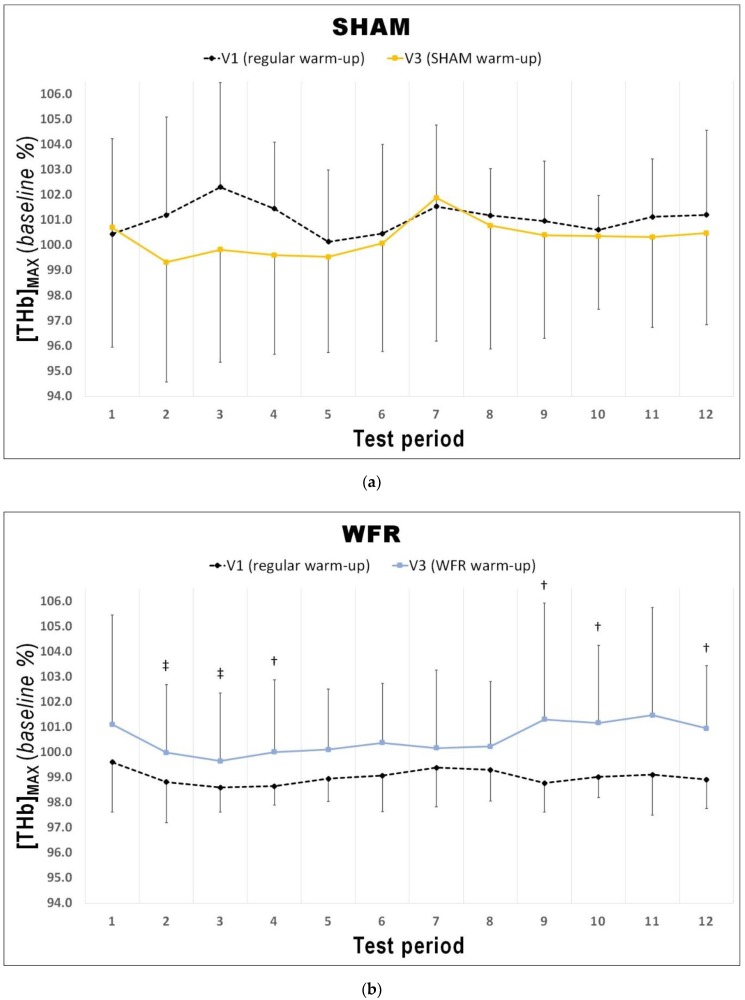
Changes in [THb]_MAX_ from V1 to V3 during each period of the RSA test under (**a**) SHAM and (**b**) WFR conditions. Note: Data are expressed as percentage of the resting baseline values and presented as means ± SD. WFR compared to SHAM: †, likely large effect; ‡, very likely large effect.

**Figure 4:**
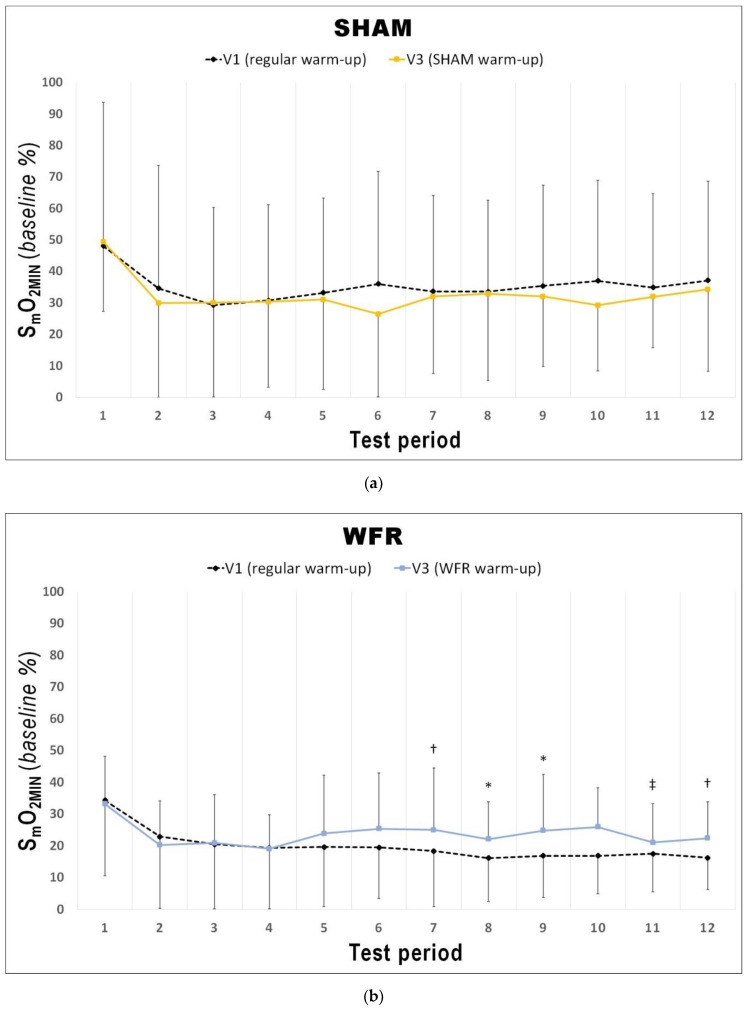
Changes from V1 to V3 in S_m_O_2MIN_ during each period of the RSA test under (**a**) SHAM and (**b**) WFR conditions. Note: Data are expressed as percentage of the resting baseline values and presented as means ± SD. WFR compared to SHAM: *, possibly small effect; †, likely small effect; ‡, likely moderate effect.

**Figure 5:**
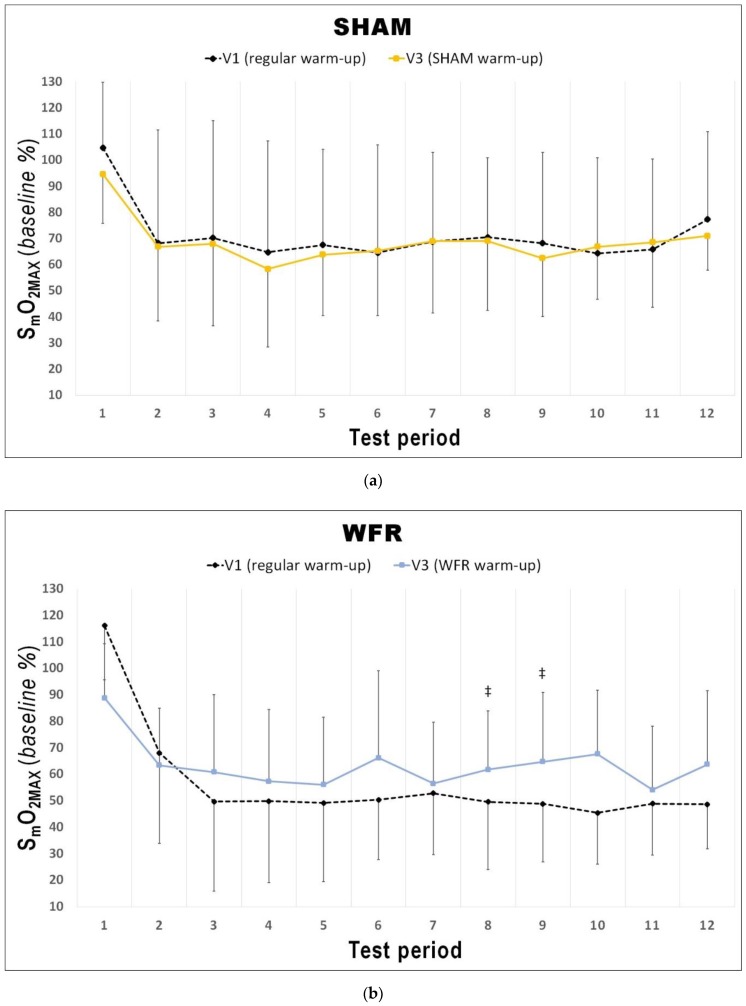
Changes from V1 to V3 in S_m_O_2MAX_ during each period of the RSA test under (**a**) SHAM and (**b**) WFR conditions. Note: Data are expressed as percentage of the resting baseline values and presented as means ± SD. WFR compared to SHAM: ‡, likely moderate effect.

Changes in average S_m_O_2_ and [THb] are presented in Table 2 and Figure 3, Figure 4 and Figure 5. During the warm-up, the impediment of blood flow did not induce any meaningful changes in [THb]_AVG_ and S_m_O_2AVG_. During the RSA test, however, although no change was reported for S_m_O_2AVG_, there was a tendency for greater [THb]_AVG_ after WFR (ΔV3−V1: 1.5% difference, ES 0.46 ± 0.81, 71/20/9%).

The changes in [THb]_MAX_ for each period of the RSA tests in WFR and SHAM are displayed in Figure 3. WFR had a meaningful effect on local blood volume for 6 of the 12 periods. Indeed, the experimental warm-up produced a very likely large positive effect on [THb]_MAX_ in periods 2 and 3, and a likely large positive effect in periods 4, 9, 10, and 12 (period 2: ES 1.25 ± 0.92, 97/2/1%; period 3: ES 1.43 ± 0.98, 98/2/1%; period 4: ES 1.31 ± 1.19, 94/4/2%; period 9: ES 1.25 ± 1.43, 89/6/5%; period 10: ES 0.98 ± 1.11, 88/8/4%; period 12: ES 1.12 ± 1.28, 89/6/5%). WFR further increased S_m_O_2MIN_ and S_m_O_2MAX_ during the RSA test (Figure 4 and Figure 5). Specifically, the experimental warm-up produced a likely moderate effect on S_m_O_2MIN_ in period 10, a likely small effect in periods 7 and 12 and a possible small effect in periods 8 and 9 (period 7: ES 0.47 ± 0.62, 78/18/4%; period 8: ES 0.37 ± 0.52, 72/25/4%; period 9: ES 0.34 ± 0.51, 69/27/4%; period 10: ES 0.54 ± 0.46, 89/10/1%; period 12: ES 0.43 ± 0.24, 94/6/0%). WFR had a likely moderate effect on S_m_O_2MAX_ in periods 8 and 9 (period 8: ES 0.51 ± 0.64, 80/17/4%; period 9: ES 0.57 ± 0.53, 88/10/1%).

## 4. Discussion

The current study aimed to determine whether performing a warm-up with impeded blood flow could improve local blood volume, muscle oxygenation, and repeated-sprint ability. The major findings were that applying a moderate pressure (7 out of 10 on a 0-to-10 rating scale) with elastic wraps on both thighs for ≈ 15 minutes during warm-up clearly increased local blood volume, attenuated muscle deoxygenation, and enhanced muscle reoxygenation during some parts of a subsequent RSA test more than a usual warm-up in trained American football players. However, these physiological responses to WFR did not meaningfully enhance performance of twelve 20 m sprints.

Vascular compression during exercise induces significant changes in local blood volume. Yanagisawa and Fukutani demonstrated that the total hemoglobin/myoglobin concentration significantly increases during exercise with BFR [36], likely because of the impeded venous outflow. Along this line of reasoning, Willis and colleagues reported greater positive changes in tissue perfusion (via blood volume, Δ [THb]) during repeated cycling sprints with BFR compared to non-restricted repeated sprints [46]. Performing BFR during exercise also appears to affect muscle oxygenation. It has been shown that the tissue oxygenation index (TOI) significantly decreases during exercise with vascular compression performed at 158 mmHg [36,47]. In our study, there was no clear difference between SHAM and WFR for average values of [THb] and S_m_O_2_ during the first section of the warm-up, while moderate pressure was applied on the participants’ lower limbs (Table 2). One could argue that the pressure used in our experimental protocol was not high enough to alter muscle hemodynamics and oxygenation, but when comparing thigh volumes measured pre- and post-compression at V3, WFR readily increased limb girth (Table 1), which suggests venous blood pooling in the lower limb. It is possible that the NIRS device used in this study was not precise enough to detect such changes in blood volume (see limitations discussion below). Nonetheless, this WFR-induced transient increase in thigh volume is concordant with the findings of Wilson and colleagues regarding the impact of BFR on overall muscle thickness [41], and indicates that a moderate pressure of 7/10 was enough to produce meaningful physiological alterations that could account for the changes in [THb] and S_m_O_2_ observed post-compression.

In fact, during the RSA test, [THb]_MAX_ was greater after WFR than SHAM for 6 of the 12 periods (Figure 3). This is in accordance with studies showing the influence of BFR on muscle reactive hyperemia post-compression [36]. This enhanced muscle perfusion may be caused by the acute release of NO [33] and adenosine [34], two substances with powerful vasodilatory properties. Considering that greater muscle blood volume increases O_2_ availability to the tissues [48], we could expect skeletal muscle oxygenation to change. Not surprisingly then, WFR elevated S_m_O_2MIN_ in 5 of the 12 periods of the RSA test concomitantly to [THb]_MAX_ values. These data demonstrate a greater muscle oxygenation during maximal efforts, and probably indicate a greater O_2_ delivery to the muscle during these periods. It is also interesting to note that some S_m_O_2MIN_ were elevated despite no clear concomitant change in the corresponding [THb]_MAX_ values (i.e., periods 7 and 8). It is generally accepted that improving the contribution of oxidative phosphorylation to energy production may minimize fatigue during RSE. An elevated aerobic metabolism may spare the utilization of the finite anaerobic energy stores, reduce the accumulation of fatigue-related metabolites, and accelerate phosphocreatine resynthesis between sprints [6,49]. In addition, greater local blood volume may allow for a faster removal of metabolic waste products during periods of recovery [50]. Given that participants from the WFR group were able to maintain the same overall performance at V3 compared to V1 despite no change or even higher S_m_O_2MIN_, we could speculate that WFR may improve the metabolic efficiency of muscle contraction. This assumption appears supported by observations made in a previous study involving a compression protocol that allows manipulating blood flow to skeletal muscles. Paradis-Deschênes and colleagues [51] reported higher force developed during maximal knee extensions despite lower average O_2_ uptake following the use of ischemic preconditioning.

Since muscle reoxygenation during recovery periods between sprints has been demonstrated as a physiological determinant of RSA [16], the current NIRS analysis also examined the reoxygenation status of the muscle after WFR. Another novel finding of the current study was that performing a warm-up with impeded blood flow increased S_m_O_2MAX_ in 2 of the 12 periods of the RSA test (i.e., periods 8 and 9). Buchheit and Ufland were the first to report the link between post-sprint muscle reoxygenation rate and RSA improvement [17], which directly reflects that phosphocreatine resynthesis is almost exclusively O_2_ dependent [52]. This observation is also consistent with several other studies suggesting that muscle aerobic function might play a role in the metabolic recovery between sprints [7,8,9,10,11,15,16].

We originally reasoned that because WFR could promote vasodilation and O_2_ delivery, a meaningful impact on performance would be observed during a subsequent exercise that impedes blood flow to the skeletal muscles. However, WFR did not significantly improve RSA under the conditions of our study. On one hand, we could speculate that the intensity of the warm-up was already high enough, without compression, to induce positive changes on performance, which led to trivial differences between WFR and SHAM. On the other hand, we could argue that our RSA test was too short to observe clear differences. According to studies on aerobic contribution to RSE, athletes normally reach their VO_2_max during later sprints [13,53]. It has also been shown that better O_2_ delivery to the muscles may accelerate VO_2_ kinetics when exercise intensity reaches VO_2_max [21,22,23]. Collectively, these observations suggest that the enhanced blood volume ([THb]_MAX_) along with elevated muscle O_2_ saturation (S_m_O_2MIN/MAX_) measured toward the end of the RSA test would have affected performance more importantly with additional sprints. Future research should investigate the use of longer sprint protocols and/or actual games to further explore the potential ergogenicity of WFR on reoxygenation capacity and performance. Furthermore, since many team sports can be played at an altitude sufficient to challenge the oxidative metabolism, implementing the current new warm-up could provide ergogenic benefits to some players (in particular those exhibiting the largest arterial desaturation) and this approach should be tested.

The current results are to be considered in light of the following technical limitations. An important consideration when using vascular compression is the minimal restrictive pressure applied to the limb required to impede the venous outflow and reduce the arterial inflow. While a perceived pressure of 7/10 has been shown to consistently result in complete occlusion of the veins, but not the arteries [41], more recent data do not demonstrate large differences in ratings of discomfort during exercise across a variety of pressures [32], suggesting that participants may need several familiarization sessions before being able to successfully estimate the actual pressure. Most of the participants in the current study were new to the blood-flow manipulation technique and they probably would have needed more than one session to get accustomed to the rating scale. Although we are confident that the pressure applied on the participants’ lower limbs induced a significant physiological response, larger muscle hemodynamics and oxygenation alterations might have been observed if the participants were more accustomed to the method. Finally, the NIRS device used may not be accurate enough to detect small changes in hemodynamics and oxygenation. Crum and colleagues reported no change in the MOXY [THb] variable across varying intensities [54]. However, [THb] exhibited strong reliability between tests (CV ≤ 1%). The authors also reported a lower reliability for the MOXY S_m_O_2_ at high intensities (power output ≤150 W, CV ≤ 5% vs. power output ≥200 W, CV ≥ 12%). Therefore, the hemodynamics changes reported here will have to be ascertained to better comprehend the efficacy of this new approach to the warm-up.

## 5. Conclusions

The present study suggests that performing ≈15 min of warm-up with blood-flow restriction at a moderate perceived pressure of 7 out of 10 may increase local blood volume ([THb]_MAX_) and muscle O_2_ saturation (S_m_O_2MIN/MAX_) in highly trained American football players during some parts of a subsequent RSA test. Although this innovative WFR approach did not clearly impact the performance of 12 sprints or less, the altered physiological responses could prove beneficial to American football players and other team-sport athletes in longer activities involving multiple bouts of maximal efforts.

## Figures and Tables

**Table 1:** Thigh volume measured pre- and post-compression during visit 3 (V3) under regular warm-up (SHAM) and warm-up combined with blood-flow restriction (WFR) conditions.

	Pre-Compression (dL)	Post-Compression (dL)	% Difference Post vs. Pre	ES (90% CL)	% Chances for WFR to Be ↗ / = / ↘ than SHAM
**SHAM**	60.3 ± 9.9	60.4 ± 9.2	0.2 ± 2.2	0.21 (0.07, 0.36)	57/43/0
**WFR**	57.3 ± 5.6	59.0 ± 6.0	3.0 ± 1.0 *		

Note: Data are presented as means ± SD. WFR compared to SHAM: *, possibly small effect.

**Table 2:** Average muscle oxygenation variables collected during first section of the warm-up and repeated-sprint ability (RSA) test under SHAM and WFR conditions.

	SHAM			WFR			ES (90% CL)	% Chances for WFR to Be ↗/=/↘ than SHAM
	V1	V3	ΔV3−V1	V1	V3	ΔV3−V1		
**1st section warm-up**								
**S_m_O_2avg_**	85.9 ± 17.4	77.5 ± 21.3	−8.5 ± 5.3	77.4 ± 16.4	66.3 ± 21.5	−11.1 ± 11.5	−0.06	0/96/4
							(−0.19, 0.07)	
**[THb]_avg_**	99.0 ± 1.6	99.4 ± 1.2	0.3 ± 1.6	98.8 ± 1.2	99.6 ± 1.0	0.8 ± 1.7	0.18	47/40/12
							(−0.38, 0.73)	
**RSA test**								
**S_m_O_2avg_**	52.7 ± 32.8	50.4 ± 23.3	−2.3 ± 12.8	36.7 ± 16.5	42.5 ± 18.8	5.9 ± 18.7	0.15	41/52/7
							(−0.24, 0.54)	
**[THb]_avg_**	98.8 ± 2.5	98.1 ± 2.8	−0.6 ± 2.9	97.8 ± 1.2	98.7 ± 2.1	0.9 ± 2.5	0.46	71/20/9
							(−0.35, 1.27)	

Note: Data are presented as means ± SD and expressed as percentage of the resting baseline values.
